# Basophils activation of patients with chronic spontaneous urticaria in response to C5a despite failure to respond to IgE-mediated stimuli

**DOI:** 10.3389/fimmu.2022.994823

**Published:** 2022-09-28

**Authors:** Daiki Matsubara, Yuhki Yanase, Kaori Ishii, Shunsuke Takahagi, Akio Tanaka, Koichiro Ozawa, Michihiro Hide

**Affiliations:** ^1^ Department of Dermatology, Graduate School of Biomedical and Health Sciences, Hiroshima University, Hiroshima, Japan; ^2^ Department of Pharmacotherapy, Graduate School of Biomedical and Health Sciences, Hiroshima University, Hiroshima, Japan; ^3^ Department of Dermatology, Hiroshima City Hiroshima Citizens Hospital, Hiroshima, Japan

**Keywords:** peripheral basophils, IgE, complement, chronic spontaneous urticaria (CSU), histamine

## Abstract

Urticaria is characterized by the occurrence of wheals and flares in response to vasoactive mediators, such as histamine. Various studies have suggested the involvement of basophils in the pathogenesis of chronic spontaneous urticaria (CSU). However, histamine release from peripheral basophils in response to stimuli acting on the high affinity IgE receptor (FcϵRI) is impaired in many patients with CSU (non/low responders). We previously demonstrated that tissue factor (TF)s expressed on vascular endothelial cells in response to a combination of various stimuli, such as that of histamine and lipopolysaccharide (LPS), activates the extrinsic coagulation pathway and produces anaphylatoxin, complement 5a (C5a), which then activates basophils and mast cells *via* the C5a receptor (C5aR). We have revealed that histamine release was induced in response to C5a and formyl-l-methionyl-l-leucyl-l-phenylalanine (fMLP), regardless of the response to anti-IgE antibody, the reduced numbers of basophils and severity of urticaria. Moreover, we found that spontaneous release of histamine *ex vivo* from basophils of patients with CSU is higher than that from healthy individuals. These results suggest that basophils and the complement system, which could be activated by coagulation factors, may play a critical role in the pathogenesis of CSU, especially in cases refractory to treatment involving the IgE/FcϵRI pathway.

## Introduction

Chronic spontaneous urticaria (CSU), also called as chronic idiopathic urticaria (CIU), is a skin disorder characterized by daily or almost daily recurring wheals and flares, with itch occurring anywhere on the body for more than 6 weeks. The formation of wheals and flares are induced by chemical mediators, especially histamine, which may be released from mast cells and basophils (1). Generally, basophils and mast cells express high affinity IgE receptors (FcϵRIs) on the plasma membrane surface and bind antigen-specific IgEs to FcϵRIs. When specific antigen binds to IgEs on the surface of cells, basophils and mast cells are activated and release inflammatory mediators, such as histamine, followed by an increase of vascular permeability and edema formation. Several reports suggest that 30-50% of patients with CSU have IgG autoantibodies against IgE antibody and/or FcϵRI (2, 3). Moreover, IgE autoantibodies against endogenous molecules, such as dsDNA, interleukin (IL)-24, tissue factor (TF) and thyroid peroxidase (TPO), have also been detected in a certain population of patients with CSU (3). Furthermore, basophils migrate from blood vessels into the skin during wheal formation, and are suggested to contribute to the persistence of wheals in CSU (4). Rapid effect of omalizumab, an anti-IgE monoclonal antibody for the treatment of CSU also shows the importance of FcϵRI-dependent activation of basophils rather than mast cells (5). However, the number of peripheral blood basophils and the histamine releasing activities of peripheral basophils of healthy donors and patients with CSU in response to anti-IgE antibody (anti-IgE), an activator of the IgE-FcϵRI pathway, are significantly decreased (non- or low-responder) (6) (7). These features of basophils could be explained by the activation of FcϵRI on basophils either spontaneously or by endogenous stimuli. However, histamine releasing activities of IgG antibodies against IgE and/or FcϵRI or IgE antibodies against autoantigens in patients with CSU shown *in vitro* have not been demonstrated to fully activate basophils by themselves in patients with CSU *in vivo*, even in severe cases (2, 8). Moreover, the presence of such autoantibodies is not detected in more than a half of patients with CSU, and the expression of FcϵRI on basophils may be scant especially in patients with CSU refractory to omalizumab treatment (9). Therefore, how peripheral basophils are activated and release histamine in patients with CSU, especially in non- or low-responders, has been largely unclear. To date, we have revealed a relationship between TF expression, activated coagulation factors and complement factors. Treatment of vascular endothelial cells with histamine released from human peripheral basophils or VEGF together with several proinflammatory molecules, such as lipopolysaccharide (LPS), tumor necrosis factor (TNF)α, IL-1β or IL-33, synergistically increase TF expression on endothelial cells (10). High expression of TFs on the cell surface then activates the extrinsic coagulation pathway and produces active forms of coagulation factors, such as factor (F)Xa and FIIa (thrombin), resulting in inter-cellular gap formation of vascular endothelial cells *via* protease activated receptor 1 (PAR1). Moreover, Asero and our group reported that plasma levels of prothrombin fragment 1 + 2 (PF_1+2_) and D-dimer in patients with CSU are higher compared to normal controls, and correlate with disease severities (11, 12). Furthermore, the extrinsic coagulation potential is elevated in patients with CSU (13). Of note, we revealed that an active form of complement 5 (C5a), produced by the activated coagulation factors or plasmin, induces histamine release from basophils of healthy donors and skin mast cells *via* the C5a receptor (C5aR) (3) (14). In fact, the increase of plasma C5a concentration was reported in patients with CSU (15). Moreover, several reports suggest that anticoagulant drugs, such as heparin or warfarin are effective for the treatment of CSU (16, 17). These reports imply that basophils trigger and/or promote the activation of the coagulation pathway and subsequently-produced C5a plays a major role in the pathogenesis of CSU. Previously, Zuberbier, et al. revealed that basophils of patients with CSU pre-treated with interleukin-3 (IL-3) release histamine in response to C5a, but not to anti-IgE (18). On the other hand, Luquin et al. reported that histamine release from basophils of patients who suffered from CSU without basopenia, was less than that from basophils of healthy controls (19). Moreover, Vasagar, et al. reported that basophils isolated from patients with CSU in base line conditions by density fractionation express higher in CD63 but normal in CD203c as compared with healthy controls (20). In this study, we obtained basophil-enriched leukocytes fractions from patients with CSU with different disease severity and assessed their histamine release in response to anti-IgE, C5a and formyl-l-methionyl-l-leucyl-l-phenylalanine (fMLP) stimulation in a non-IL-3 treated condition. The levels of spontaneous release of histamine from leukocyte fractions, plasma histamine, and basophil activation markers, CD203c and CD63 in patients with CSU were also compared with those of healthy controls.

## Methods

### Reagents and instrument

The chemicals used in this study were obtained from the following sources: human serum albumin (HSA) and fMLP from Sigma–Aldrich Japan (Tokyo, Japan). Anti-human IgE antibody from BETYL (Montgomery, TX). Ficoll-Paque Plus was from GE Healthcare Japan Corporation (Tokyo, Japan). Reverse-phase HPLC was from Shimadzu (Kyoto, Japan). Fluorescence labeled anti-cluster of differentiation (CD)63 antibody was from Biolegend (San Diego, CA). C5a were from R&D Systems Inc. (Minneapolis, MN). Allergenicity^®^ kit was from Beckman Coulter, Inc. (Brea, CA). Single cell fluorescence levels were analyzed by fluorescence-activated cell sorting by Attune™ Acoustic Focusing Cytometer (Life technologies, Carlsbad, CA, USA). Basophil number in the blood was counted by automated complete blood cell counter (Sysmex Japan, Tokyo, Japan)

### Measurement of histamine

Leukocytes including basophils were obtained from peripheral blood as described in our previous paper (14). Briefly, fresh blood was obtained with ethylenediaminetetraacetic acid (EDTA) from each donor by venipuncture. The whole blood was mixed with the same volume of 1% methylcellulose in saline and then allowed to stand at room temperature for 30 min. A supernatant with abundant leukocytes was collected, leaving red blood cells. The amount of histamine in or out of cells was measured by means of reverse-phase high performance liquid chromatography (HPLC) (14). Histamine release tests were performed as described previously (21) with a goat anti-IgE (670 ng/ml), C5a and fMLP at the indicated concentrations. Cells were not pretreated with IL-3 prior to the stimuli in experiments. Spontaneous release from basophils of each patient was calculated by the amount of histamine in buffer and basophils after incubation for 45 min.

### Analysis by flow cytometry

Expression levels of CD203c and CD63 on the surface of basophils were detected using Allergenicity^®^ kit according to the manufacturer’s instructions adding the anti-CD63 antibody. Briefly, whole blood cells were stained with anti-CD203c-PE and anti-CD63-APC in the presence or absence of anti-IgE or C5a at indicated concentrations for 15 min at 37°C. After incubation, red blood cells were ruptured by osmotic pressure. Fluorescence level of anti-CD203c and anti-CD63 was measured using Attune™ Acoustic Focusing Cytometer (Life technologies).

### Subjects

**Figure 1:**
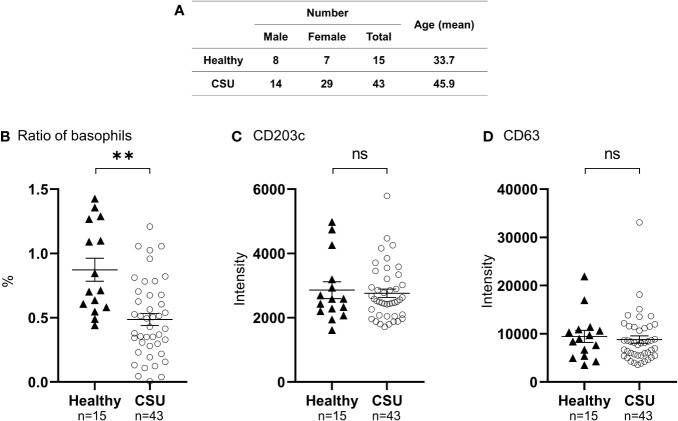
Analysis of peripheral basophil conditions by flow cytometry. **(A)** Characteristics of patients with CSU, and healthy controls. **(B)** Ratio of peripheral basophils to total white blood cells in patients with CSU and healthy donors. Five hundred basophils were detected in each measurement. Mean ± SEM of the number of basophils and white blood cells counted in the blood of 27 patients with CSU were 23.8 ± 3.00/µL, and 6911± 393, respectively. **(C, D)** Detection of basophil activation markers, CD203c and CD63, by flow cytometry analysis. The statistical difference was determined by the t-test. Differences between two groups was considered significant at **P < 0.01. ns, not significant.

**Figure 2:**
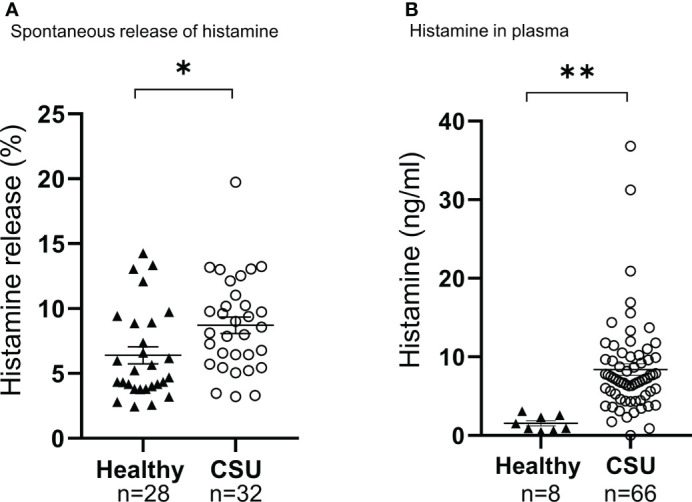
Analysis of spontaneous histamine release of basophils **(A)**, and amount of histamine in plasma **(B)**. Data represent mean ± standard error of the mean. The statistical difference was determined by the *t*-test. Differences between two groups was considered significant at *P <0.05 **P < 0.01.

**Figure 3:**
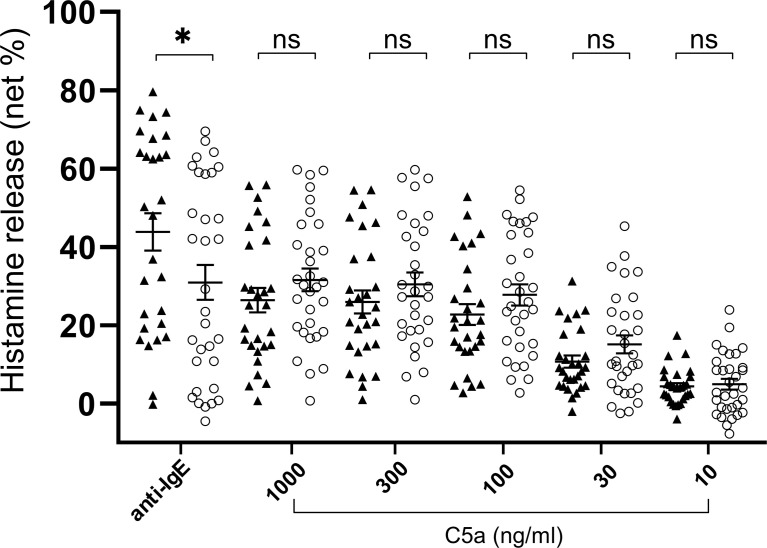
Histamine release from basophils of healthy donors (closed triangle, n=28) and patients with CSU (open circle, n=32. Only C5a 1000ng/ml, n=31). Data represent mean ± standard error of the mean. The statistical difference was determined by the T test. Difference between two groups was considered significant at *P <0.05. ns, not significant.

**Figure 4:**
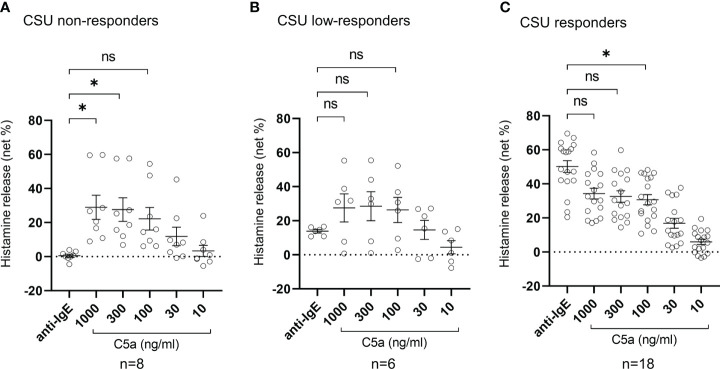
Histamine release from basophils of non-responder, low-responder, responder patients with CSU in response to anti-IgE or C5a. **(A)** CSU non-responders, **(B)** CSU low-responders, and **(C)** CSU responders. Data represent mean ± standard error of the mean. The statistical difference was determined by Tukey’s test. Difference between each group was considered significant at *P <0.05. ns, not significant.

**Figure 5:**
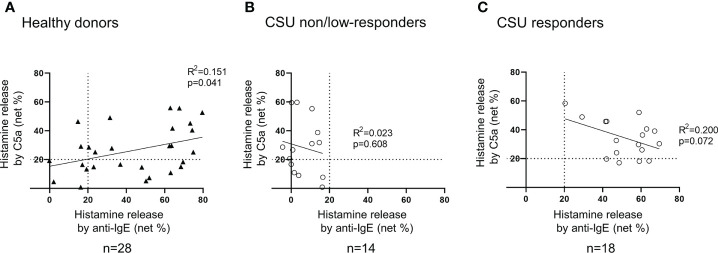
Correlation between histamine release from basophils in response to anti-IgE and that to C5a. **(A)** Healthy donors. Histamine release in response to anti-IgE and that to C5a tend to be positively correlated. **(B)** CSU non/low responders. Not more than 20% of histamine release was observed in response to anti-IgE, but various degrees of histamine release were evoked by C5a. **(C)** CSU responders. A weak tendency of a negative correlation was observed.

**Figure 6:**
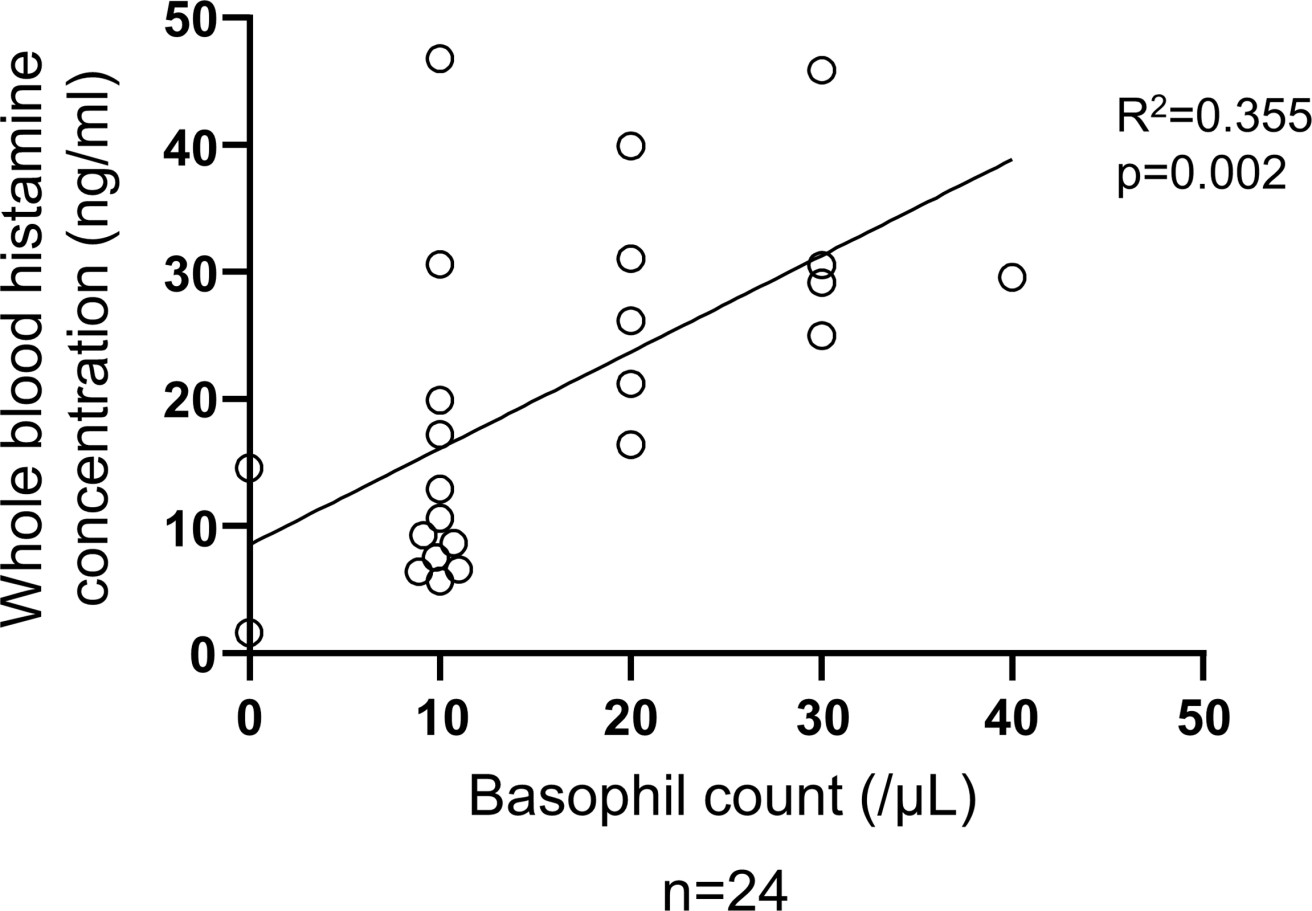
Correlation between basophil count and total histamine level in the blood of patients with CSU. Simple regression analysis revealed positive correlation between basophil count and whole blood histamine concentration (R^2^ = 0.355, P=0.002).

Blood samples were collected from patients with CSU, who visited the Department of Dermatology in Hiroshima University. Healthy volunteers were recruited with written consents to participate in this study. The study protocol was approved by the institutional ethics committee (E-1716). Demographic characteristics of donors whose samples were analyzed in **Figures 1**, **2B**, and **Supplementary Figure 2** are described in **Figure 1A**, **Table 2**, and **Supplementary Figure 2A**, respectively. Demographic characteristics of donors whose samples were analyzed in **Figures 2A**, **3**–**6**, **Supplementary Figures 1**, **3**, **4** are described in **Table 1**.

**Table 1:** Demographic characteristics of patients with CSU and healthy donors in **Figures 2A**, **3–6**, **Supplementary Figures 1, 3**, and **4**.

	Healthy	CSU
Subject number	n=28	n=32
Gender (male/female)	14/14	10/22
Age (years); mean ± SEM (range)	40.7 ± 2.46 (21-64)	45.8 ± 8.10 (13-76)
Non-responder UCT UAS7	n=2	n=8 9.00 ± 0.94 (n=5) 22 ± 0 (n=2)
Low-responder UCT UAS7	n=5	n=6 4.60 ± 1.25 (n=5) 22.75 ± 4.55 (n=4)
Responder UCT UAS7	n=21	n=18 8.07 ± 1.04 (n=14) 19.6 ± 3.94 (n=9)

### Statistical analysis

Difference among each group was tested by the *t*-test or Tukey’s test using GraphPad PRISM ver.6 (GraphPad Software, San Diego, CA).

## Results

To determine the status of peripheral blood basophils in patients with CSU, we first measured the ratio of basophils in peripheral blood leukocytes and the expression levels of cell activation markers on basophil surface by means of flow cytometry. In line with previous reports, the ratio of basophils in peripheral blood leukocytes of patients with CSU was significantly low compared to that of healthy donors. (**Figures 1A, B** **)**. However, the expression levels of CD63, a degranulation marker, and CD203c, an activation marker, on the surface of peripheral basophils of patients with CSU were not significantly increased (**Figures 1A, C, D** **)**. We then analyzed spontaneous release of histamine from basophils of healthy and CSU donors, whose information is summarized in **Table 1**. As shown in **Figure 2A**, spontaneous release of histamine from basophils of patients with CSU was slightly, but significantly higher than that from basophils of healthy donors. The degree of spontaneous release of histamine was not correlated with total amount of histamine in whole blood of patients with CSU (**Supplementary Figure 1**). The measurement of plasma histamine in another set of healthy controls and patients with CSU (**Table 2**) showed that the amount of histamine in plasma of patients with CSU was significantly higher than that of healthy controls (**Figure 2B**). We then investigated histamine release activity of basophils of healthy donors and patients with CSU in response to anti-IgE (670 ng/ml) and an anaphylatoxin, C5a, at indicated concentrations (**Figure 3**). Although peripheral basophils express both C3a receptor (C3aR) and C5aR, we previously reported that C3a induces only marginal release of histamine from peripheral basophils (14). Moreover, we have confirmed that C5aR is expressed in basophils from patients with CSU and healthy donors (**Supplementary Figure 2**). Therefore, we focused on the effect of C5a as an anaphylatoxin in comparison with anti-IgE. As shown in **Figure 3**, basophils of most healthy donors released a large amount of histamine in response to anti-IgE and C5a. However, histamine release from basophils induced by C5a is weaker than that induced by anti-IgE. Basophils of a certain population of healthy donors and patients with CSU released no or only slight amount of histamine. For convenience, we defined these patients as non- or low-responder when their histamine release from basophils was less than 5% or 20% in response to anti-IgE, respectively. The population of non- or low-responders in patients with CSU tend to be higher than that in healthy donors. However, no apparent difference in severity of urticaria was found between non-/low-responders and responders (**Table 1**). On the other hand, C5a induced histamine release from basophils of most subjects in the groups in a dose-dependent manner. Moreover, histamine release in response to C5a from basophils of patients with CSU was similar to or even higher than that of healthy donors (**Figures 3**, **4**). Of note, histamine release from basophils in response to anti-IgE and that to C5a in CSU responders tended to be negatively correlated, whereas those in healthy donors showed a tendency of positive correlation (**Figure 5**). Basophils of both responders and non-/low-responders were also activated by fMLP which activates basophils by an FcϵRI-independent pathway (**Supplementary Figure 3**). To further confirm the potential of histamine release by basophils in the blood, we analyzed the amount of total histamine and the number of basophils in the blood of patients with CSU. As shown in **Figure 6**, the level of whole blood histamine was positively correlated with the number of basophils, but it was not totally reduced even in blood with low numbers of basophils (**Figure 6**). Unexpectedly, the amount of histamine per basophil tended to be inversely correlated with the number of basophils in the blood of patients with CSU (**Supplementary Figure 4**).

**Table 2:** Characteristics of patients with CSU and healthy controls in **Figure 2B**.

	Healthy	CSU
Subject number	n=8	n=66
Gender (male/female)	4/4	25/41
Age (years); mean ± SEM (range)	31.9 ± 2.87(25-50)	37.5± 18.8 (5-90)

## Discussion

In this study, we demonstrated that basophils of low- and non-responders of CSU patients, whose basophils release low or no amount of histamine in response to anti-IgE, maintain the capacity to release histamine in response to stimuli, that is independent of the IgE-FcεRI pathway, such as C5a and fMLP. Antigen-IgE activates basophils *via* several tyrosine kinases, such as syk, and thus, non- or low-release of histamine from basophils is considered to represent a loss of function of tyrosine kinases (20). We also confirmed that the number of basophils was significantly decreased in the peripheral blood of patients with CSU.

Vasagar et al. also reported the elevation of CD63, but not of CD69 and CD203c, on the surface of basophils of patients with CSU (22). However, we found that the expression levels of both degranulation/activation markers, CD63 and CD203c, were not increased in basophils of patients with CSU. Expression levels of degranulation/activation markers of basophils may change during cell preparation. Vasagar, et al. labelled basophils isolated by double gradient fractionation, whereas we employed heparinized whole blood samples without particular cell isolation procedure. Moreover, the level of basophil activation in peripheral blood may change at the timing of blood collection due to the migration of activated basophils to dermis, and diurnal fluctuation in new basophil differentiation in the bone marrow and their emergence into the peripheral blood circulation with a lifetime of only 3-4 days (23) (24). Therefore, further studies are necessary to characterize the detailed behaviors of activation markers on peripheral basophils. Nevertheless, this study demonstrated that basophils of patients with CSU are not constitutively activated in the blood. On the other hand, both spontaneous histamine release from basophils isolated *ex vivo*, and plasma concentration of histamine of patients with CSU were higher than those of healthy donors, suggesting a certain difference of function between basophils of patients with CSU and those of healthy individuals (**Figure 2**).

Previous reports of spontaneous histamine release from basophils of patients with CSU are not consistent. Luquin, et al. reported a significant increase in patients with CSU (19), but Wahn, et al. found no difference between patients with CSU and healthy controls (25). It should be noted that both Luquin, et al. and Wahn, et al. studied pretreated basophils with IL-3 after the isolation by dextran sedimentation from the blood, whereas we isolated basophils by methyl-cellulose sedimentation and did not treat with IL-3. The real spontaneous release of histamine from basophils into the blood circulation should be a subject of future studies. Nevertheless, our results suggested that basophils of patients with CSU are susceptible to non-IgE stimuli or even to non-specific conditions to promote histamine release. Interestingly, spontaneous release of histamine (%) was not correlated to the whole blood histamine concentration (ng/ml) (**Supplementary Figure 1**), which mostly reflects the sum of the intracellular amounts of histamine in basophils (**Figure 6**) (26), but the amounts of histamine of individual basophils of patients were maintained or even higher in many patients with basopenia (**Supplementary Figure 4**). In fact, capacity to release histamine of basophils to either C5a or anti-IgE was maintained in almost all patients with basopenia as well (**Supplementary Figure 5**).

**Figure 7:**
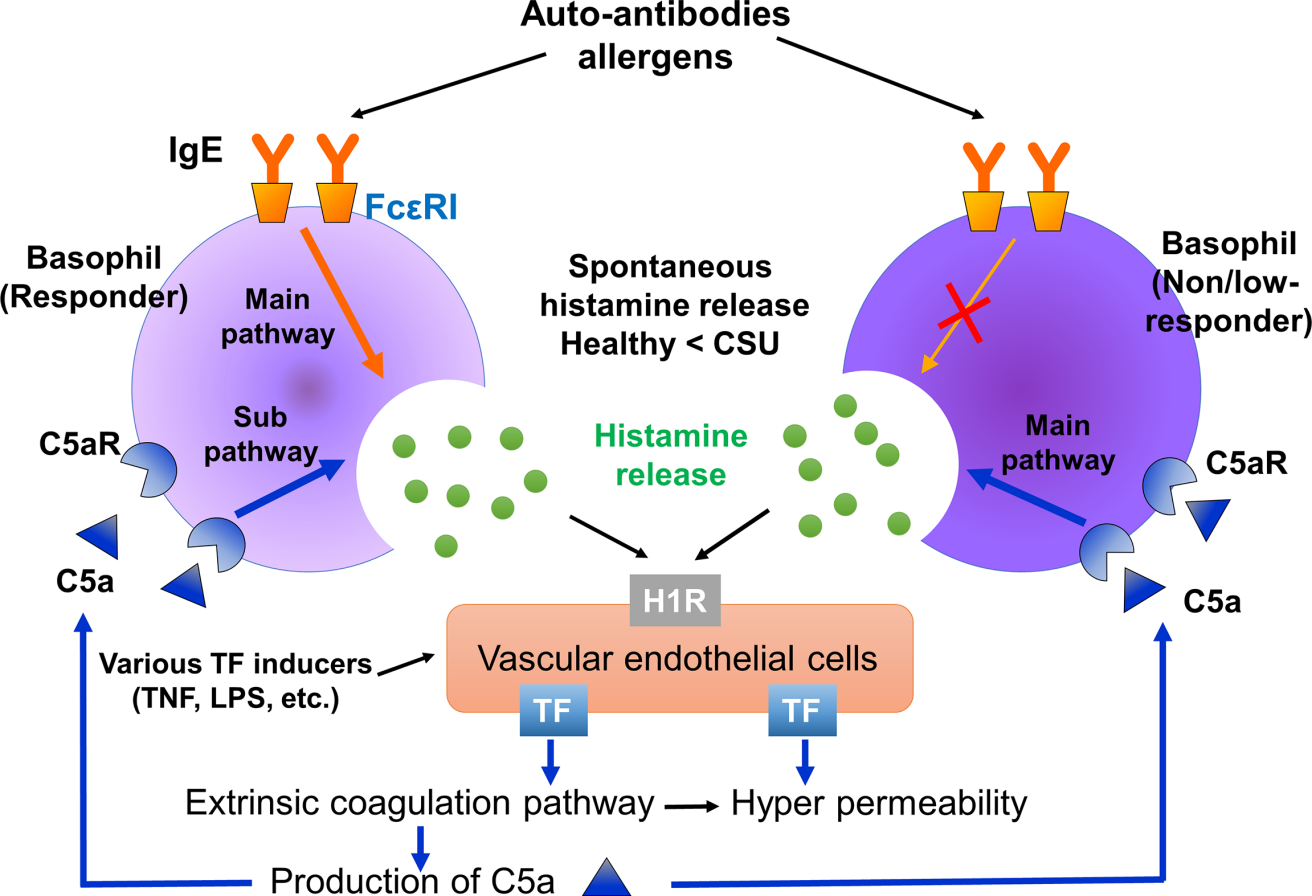
Summarized image of the role of basophils of responders and non-responders in CSU. Basophils of responders are activated *via* both the IgE-FcϵRI and C5a-C5aR pathways. On the other hand, basophils of non/low-responders are activated *via* the C5a-C5aR, but not the IgE-FcϵRI pathway. Therefore, C5a-C5aR stimulation may be a main activation pathway of non/low-responders with CSU. Histamine released from basophils may synergistically induce TF expression on vascular endothelial cells together with certain proinflammatory substances, such as LPS, TNFα. Spontaneous histamine released from basophils and other TF inducers may contribute to the synergistic expression of TF on vascular endothelial cells, followed by C5a production through the extrinsic coagulation pathway.

When histamine was released from basophils in the vicinity of vascular endothelial cells together with certain inflammatory substances, such as LPS, TNFα, IL-1β or IL-33, they synergistically induce TF expression on the vascular endothelial cells in a local area of the blood vessel. Highly-expressed TF then activates the extrinsic coagulation pathway, followed by the increase of vascular permeability induced by active forms of coagulation factors *via* PAR-1, C5a production, basophils/mast cells activation *via* C5aR and edema formation (**Figure 7**). As described above, Luquin et al., also reported the increase of spontaneous release and impairment of anti-IgE induced release of histamine from basophils of patients with CSU compared to those of healthy donors (19). However, they reported that the histamine release of basophils of patients with CSU in response to C5a was significantly lower than that of healthy donors (19). Considering possible difference of backgrounds among patients, we divided patients with CSU into 3 groups, namely, responders, low-responders, and non-responders, and activated their basophils in. the whole leukocyte fraction without separation to minimize possible mechanical and chemical damages by isolation procedures. The results of this study revealed that basophils circulating in the blood of patients with CSU may be largely impaired in the IgE-mediated pathway, and decreased in number, but preserve a high reactivity to C5a and histamine in individual cells. The underlying mechanism of loss of function in the IgE-FcεRI-syk pathway without an increase of cell activation markers, and decrease of basophils in the blood circulation remains unclear. However, the presence of patients with active CSU despite an impaired reaction to IgE-mediated stimuli suggests causative involvement of a non-IgE-FcεRI pathway in wheal formation of CSU. Thus, the basophil-C5a axis may play critical roles in the pathogenesis of CSU, especially that refractory to IgE-targeting medications, such as omalizumab.

## Conclusion

We demonstrated that basophils of patients with CSU, that release no or only little amount of histamine in response to anti-IgE, release substantial amounts of histamine in response to C5a as basophils with normal reactivity to anti-IgE. C5a produced by activated coagulation/fibrinolysis factors, such as FXa, FIIa, and plasmin may contribute to the pathogenesis of CSU, especially in patients whose basophils are impaired in the IgE-FcϵRI pathway. C5a and its related molecules, including C5aR might be an effective therapeutic target for patients with CSU including those that are refractory to IgE-targeting medications.

## Data availability statement

The original contributions presented in the study are included in the article/**Supplementary Materials**. Further inquiries can be directed to the corresponding authors.

## Ethics statement

The studies involving human participants were reviewed and approved by the institutional review board of Hiroshima University Hospital approved the study protocol (approval number: E-1716). Written informed consent to participate in this study was provided by the participants’ legal guardian/next of kin.

## Author contributions

DM, YY, KI, ST, AT, KO and MH designed the study and wrote the manuscript. DM, YY, KI and MH contributed to data collection. All authors contributed to the article and approved the submitted version.

## Funding

This work was partially supported by grants to YY from Takeda Science Foundation, JST CREST Grant (JPMJCR2111), Japan and Grant-in-Aid for Scientific Research (C) (21K08301) and to MH from Grant-in-Aid for Scientific Research (C)(18K08298).

## Acknowledgments

We wish to thank Dr Faiz Kermani for his manuscript review.

## Conflict of interest

MH received research grants from Eisai, GlaxoSmithKline, Kaken Pharmaceutical, Kyowa-Kirin, Mitsubishi Tanabe, Novartis, Sanofi, and Taiho Pharmaceutical, honoraria from Kaken Pharmaceutical, Kyorin Pharmaceutical, Kyowa-Kirin, Mitsubishi Tanabe, MSD, Novartis, Sanofi, Taiho Pharmaceutical, Teikoku Seiyaku, and Uriach.

The remaining authors declare that the research was conducted in the absence of any commercial or financial relationships that could be construed as a potential conflict of interest.

## Publisher’s note

All claims expressed in this article are solely those of the authors and do not necessarily represent those of their affiliated organizations, or those of the publisher, the editors and the reviewers. Any product that may be evaluated in this article, or claim that may be made by its manufacturer, is not guaranteed or endorsed by the publisher.

## Abbreviations

CSU, chronic spontaneous urticaria; FcϵRI, the high affinity IgE receptor; TF, tissue factor; LPS, Lipopolysaccharide; C5a, complement 5a; C5aR, C5a receptor;; fMLP, formyl-l-methionyl-l-leucyl-l-phenylalanine; CIU, chronic idiopathic urticaria; IL, interleukin; TPO, thyroid peroxidase; TNF, tumor necrosis factor; F, factor; PAR1, protease activated receptor 1; PF_1+2, _prothrombin fragment 1 + 2; HSA, human serum albumin; CD, cluster of differentiation; EDTA, ethylenediaminetetraacetic acid; HPLC, high performance liquid chromatography; AD, atopic dermatitis; 7-day Urticaria Activity Score, UAS7; urticaria control test, UCT; NS, not significant.
