# Supplementary figures and images for: Basophils activation of patients with chronic spontaneous urticaria in response to C5a despite failure to respond to IgE-mediated stimuli
Source: Front Immunol. 2022 Sep 28;13:994823. doi: 10.3389/fimmu.2022.994823 (PMC9559203; doi:10.3389/fimmu.2022.994823)

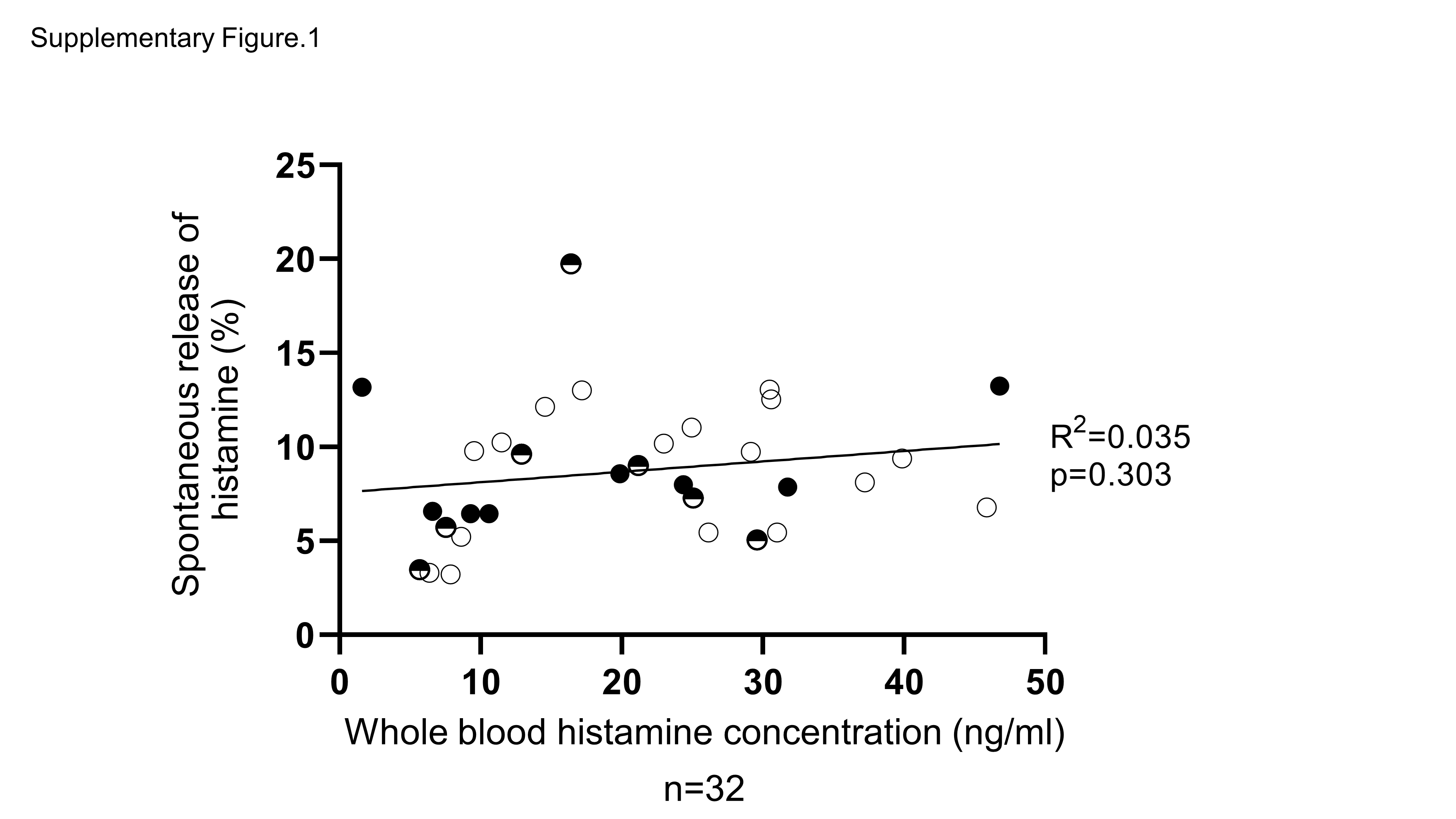

Supplement: Supplementary file 1 [file Image_1.tif]

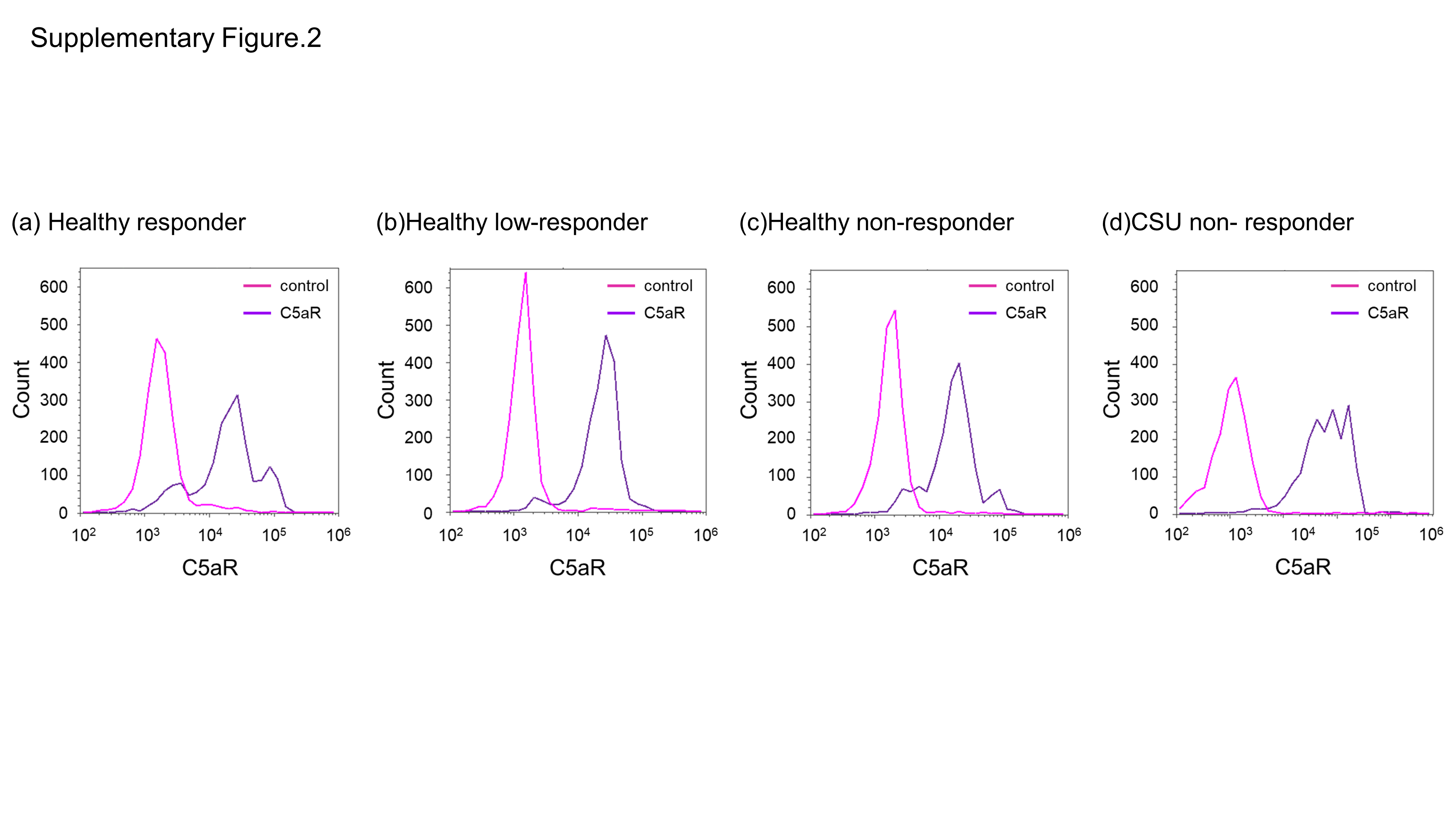

Supplement: Supplementary file 2 [file Image_2.tif]

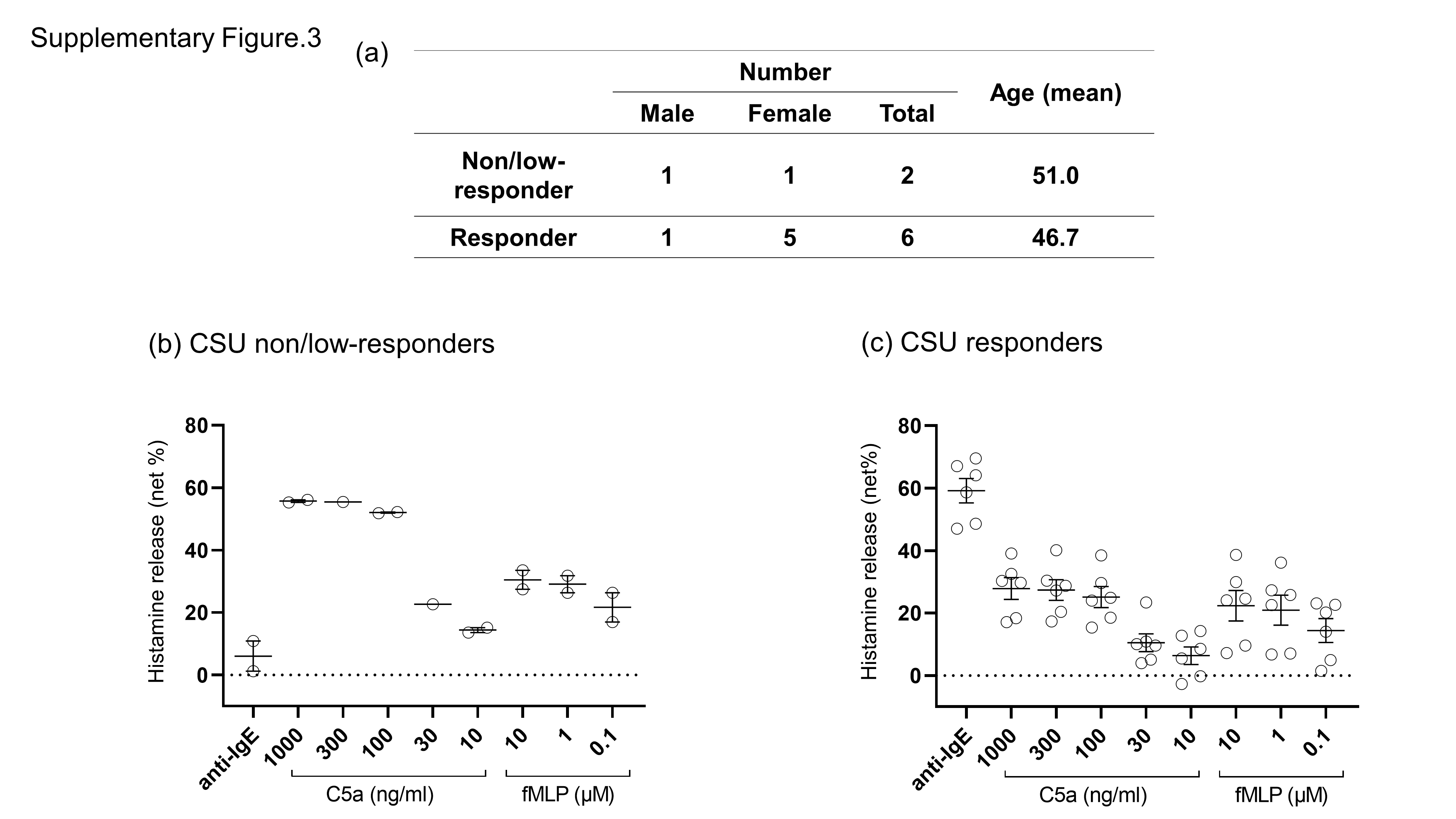

Supplement: Supplementary file 3 [file Image_3.tif]

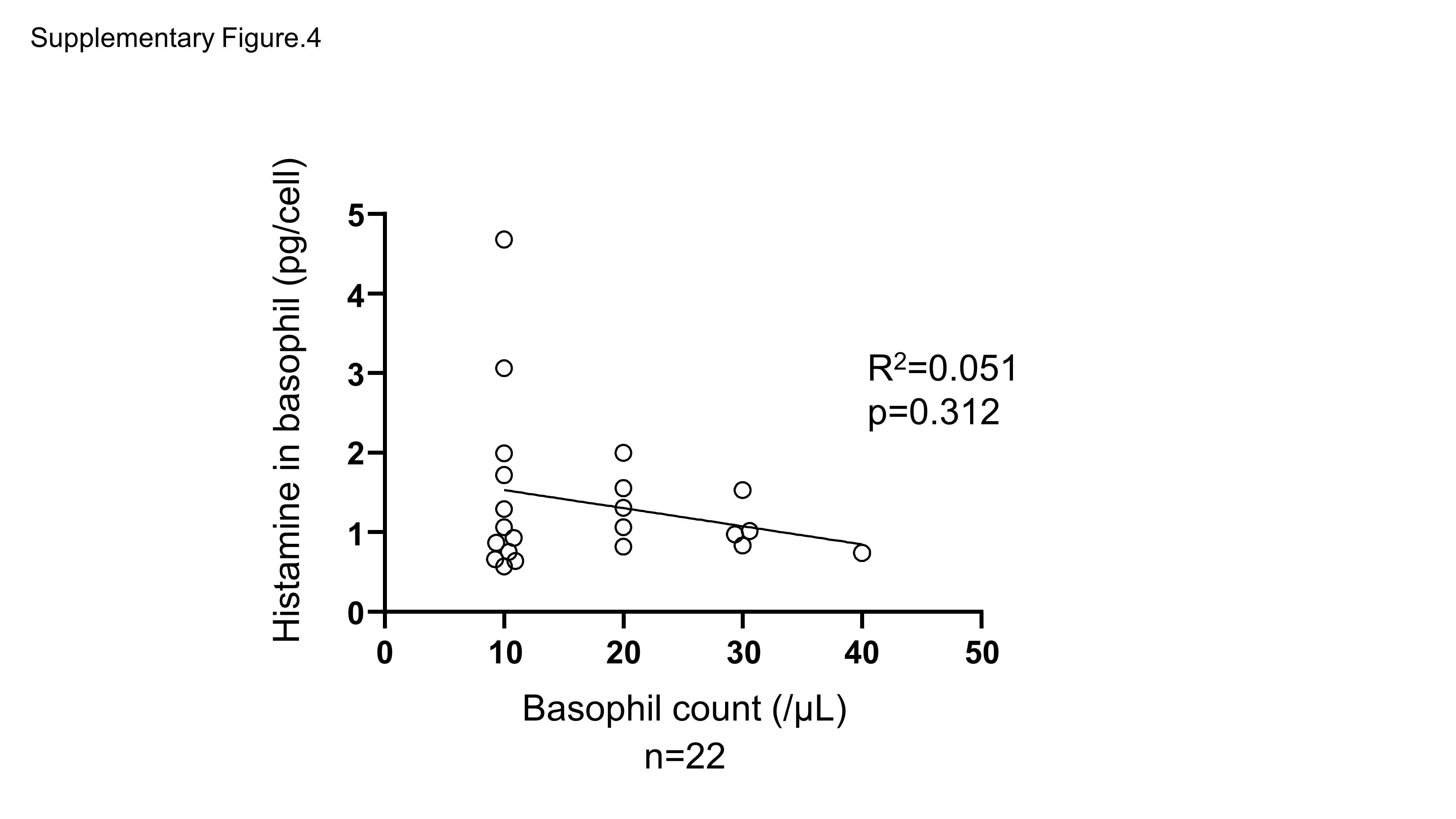

Supplement: Supplementary file 4 [file Image_4.tif]

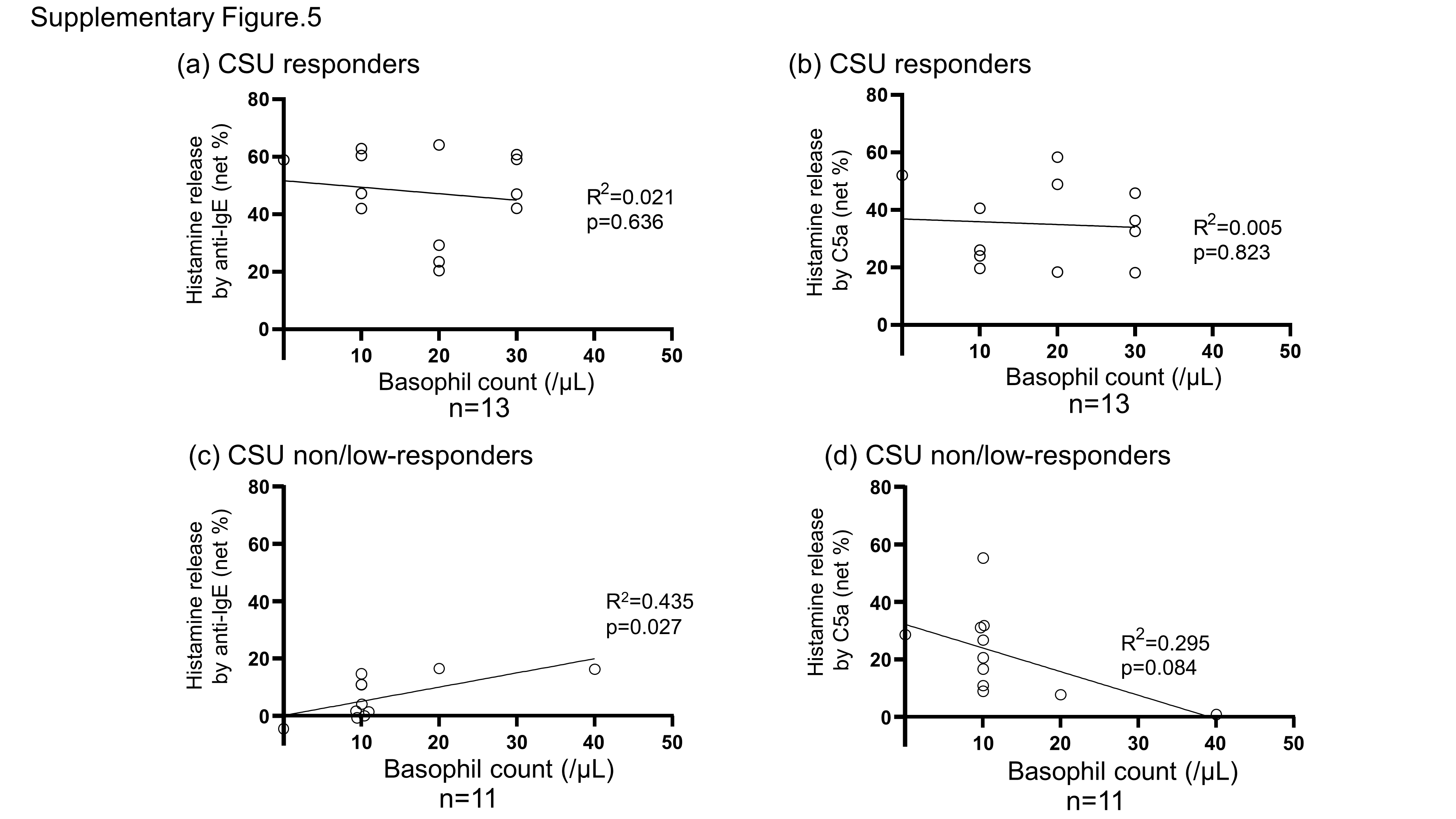

Supplement: Supplementary file 5 [file Image_5.tif]
